# Seed biopriming with P- and K-solubilizing *Enterobacter hormaechei* sp. improves the early vegetative growth and the P and K uptake of okra (*Abelmoschus esculentus*) seedling

**DOI:** 10.1371/journal.pone.0232860

**Published:** 2020-07-09

**Authors:** Muhamad Aidilfitri Mohamad Roslan, Nurzulaikha Nadiah Zulkifli, Zulfazli M. Sobri, Ali Tan Kee Zuan, Sim Choon Cheak, Nor Aini Abdul Rahman

**Affiliations:** 1 Department of Bioprocess Technology, Faculty of Biotechnology and Biomolecular Sciences, Universiti Putra Malaysia, Serdang, Selangor, Malaysia; 2 Department of Land Management, Faculty of Agriculture, University Putra Malaysia, Serdang, Selangor, Malaysia; 3 Research and Development Center, Sime Darby Plantation Research Sdn. Bhd., Carey Island, Selangor, Malaysia; Bahauddin Zakariya University, PAKISTAN

## Abstract

Limited information is available that seed biopriming with plant growth-promoting *Enterobacter* spp. play a prominent role to enhance vegetative growth of plants. Contrary to *Enterobacter cloacae*, *Enterobacter hormaechei* is a less-studied counterpart despite its vast potential in plant growth-promotion mainly through the inorganic phosphorus (P) and potassium (K) solubilization abilities. To this end, 18 locally isolated bacterial pure cultures were screened and three strains showed high P- and K-solubilizing capabilities. Light microscopy, biochemical tests and 16S rRNA gene sequencing revealed that strains 15a1 and 40a were closely related to *Enterobacter hormaechei* while strain 38 was closely related to *Enterobacter cloacae* (Accession number: MN294583; MN294585; MN294584). All *Enterobacter* spp. shared common plant growth-promoting traits, namely nitrogen (N_2_) fixation, indole-3-acetic acid production and siderophore production. The strains 38 and 40a were able to produce gibberellic acid, while only strain 38 was able to secrete exopolysaccharide on agar. Under *in vitro* germination assay of okra (*Abelmoschus esculentus*) seeds, *Enterobacter* spp. significantly improved overall germination parameters and vigor index (19.6%) of seedlings. The efficacy of root colonization of *Enterobacter* spp. on the pre-treated seedling root tips was confirmed using Scanning Electron Microscopy (SEM). The pot experiment of bioprimed seeds of okra seedling showed significant improvement of the plant growth (> 28%) which corresponded to the increase of P and K uptakes (> 89%) as compared to the uninoculated control plants. The leaf surface area and the SPAD chlorophyll index of bioprimed plants were increased by up to 29% and 9% respectively. This report revealed that the under-explored species of P- and K-solubilizing *Enterobacter hormaechei* sp. with multiple plant beneficial traits presents a great potential sustainable approach for enhancement of soil fertility and P and K uptakes of plants.

## 1 Introduction

The application of sizable inputs of synthetic chemical fertilizer such as those containing inorganic phosphorus (P) and potassium (K) has long been employed in conventional agriculture practices. While the outcome of this approach has been an inextricable success, the excessive application of chemical fertilizer particularly by large scale farming has posed detrimental impacts on environmental quality and economic growth. The plausible long-term risks and threats inflicted by the profuse usage of chemical fertilizer are still limited [1]. However, there have been several reported cases of groundwater and waterways contamination due to nutrient leaching of P, K and Nitrogen (N), especially in the areas which received high rainfalls and near streams or drains [2–5]. Such environmental issues have been extensively discussed as to mitigate the loss of nutrients added to the soil and the risk of eutrophication occurring in the adjacent water bodies. The exploration of alternative strategies that can promote competitive crop growth while maintaining environmental safety is thus a timely measure.

Recently, the application of plant growth-promoting bacteria (PGPB) has shown a promising practice for sustainable agricultural production while imposing cutbacks up to 50% on the required chemical fertilizer dose into the soils [1,6,7]. In particular, PGPB exerts beneficial effects on plant growth through several ways: facilitating the acquisition of soil nutrients by asymbiotic N_2_-fixation and inorganic P and K solubilization; modulating the level of phytohormone such as indole-acetic acid (IAA), cytokinins, and gibberellins (GA3); resisting against phytopathogen [1]. At present, majority of field-tested bacterial taxonomic groups affiliated to PGPB belonged to ranges of genera: *Azospirillum*, *Rhizobium*, *Agrobacterium*, *Rhizobium* (Alphaproteobacteria); *Azoarcus*, *Burkholderia*, (Betaproteobacteria); *Acinetobacter*, *Enterobacter*, *Klebsiella*, *Pantoea*, *Pseudomonas*, *Serratia*, (Gammaproteobacteria); *Bacillus*, *Frankia* (gram-positive bacteria) [7–10].

There are a number of documented species of *Enterobacter* which have demonstrated multiple plant growth-promoting (PGP) traits. For instance, the P solubilizing and IAA producing *Enterobacter ludwigii* has been reported to improve the root surface area and the overall growth of flax culture [11]. Whereas in the growth of chickpea and pigeonpea, *E*. *ludwigii* particularly increased the iron, zinc, copper, manganese, and calcium uptake of the harvested grains [12]. *Enterobacter cloacae* is another popular example of PGP *Enterobacter* which is ubiquitous in soil, sewage, water, and food. The efficiency of endophytic *E*. *cloacae*’s application on crop productivity has been discussed extensively in the previous reports which encompassed its functional phenotypic, genotypic and metabolomic aspects of the species [6,13–15]. Interestingly, the PGP potential of its closed counterpart, *Enterobacter hormaechei* has been recently explored. *E*. *hormaechei* has been reported to serve effectively as a biological fungicide against *Fusarium* spp. [16] and significantly improved the biosynthesis of aloin-A in aloe vera [17]. However, information that is available concerning the PGP potential of this species is scarce and further investigation is required to understand the biological activities and soil-plant-interactions involved.

Among the established techniques to introduce PGPB to plants include seed treatment, soil amendment, foliar spray, drip irrigation and root dipping in the bacterial suspensions. These techniques have certain limitations when it comes to efficiency in seed emergence, plant growth and yield quality apart from the distinctive prerequisite technical aspects as described by Mahmood et al. [18]. While there are myriads of techniques to introduce PGPB to crops, seed biopriming is one of the preferable methods to perform effective seed surface bacterial inoculation. Biopriming is conceptualized as a technique of seed priming using living bacterial inoculum which allows bacterial adherence and acclimatization to the seeds in the prevalent conditions [18]. Seed biopriming provides a lot of benefits to plants especially in enhancing seed viability, germination, seed vigor, growth, and yield [19]. Bioprimed seeds have been proven to significantly enhance the germination percentage and the germination rate of seeds even under an induced environmental stress circumstance such as osmotic stress conditions [20].

Keeping in view the importance of growth-limiting nutrient for plants such as P and K, the present study was designed to screen and characterize P- and K-solubilizing bacteria from our bacterial culture collection, retrieved from our previous bacterial isolation work on oil palm empty fruit bunch (OPEFB) and chicken manure co-composting process [21]. Additionally, the selected strains were screened for various PGP properties such as N_2_ fixation, indole-3-acetic acid production (IAA), gibberellic acid (GA3) production, zinc solubilization, siderophore and exopolysaccharide (EPS) production. To assess the effects of seed biopriming with the selected strains on okra seedling growth performance, seed germination bioassay and pot experiment under net house conditions were performed thereafter.

## 2 Materials and methods

### 2.1 Preliminary assay of qualitative P and K solubilization

Eighteen bacterial strains were revived from glycerol stock stored in –80°C freezer in Research Laboratory 1.6, Bioprocessing and Biomanufacturing Research Center, Faculty of Biotechnology and Biomolecular Sciences, Universiti Putra Malaysia. The bacterial strains were previously isolated from several sources, such as from agricultural soil, oil palm empty fruit bunch (OPEFB) and chicken manure co-composting process [21]. The bacterial strains were grown on nutrient agar at 30°C for 24 h and routinely subcultured prior to every assay.

P solubilization activity of the bacterial strains was evaluated using National Botanical Research Institute's Phosphate growth medium (NBRIP) which contains (per Liter): 10 g glucose; 5 g Ca_3_(PO_4_)_2_; 5 g MgCl_2_·6H_2_O; 0.25 g MgSO_4_·7H_2_O; 0.2 g KCl, 0.1 g (NH_4_)_2_SO_4_ and 15 g agar [22]. A modified NBRIP medium was prepared separately by the addition of a pH indicator of 10 mL bromothymol blue which contained a 0.5% aqueous solution dissolved in 0.2 N KOH [23]. Plate assay for K solubilization was performed using Aleksandrov agar (Himedia) containing insoluble K aluminosilicate. A modified Aleksandrov agar was prepared separately with the addition of 0.018 g/L phenol red dye as a pH change indicator [24]. A fresh 24 h colony culture was stabbed on the solidified agar using a sterile inoculating needle and the plate was incubated at 30°C for 5 days in triplicate. The halo zone formation was interpreted as solubility index (SI) which from now onwards was termed as P solubility index (PSI) and K solubility index (KSI) and determined using the ratio of the total halo diameter to the colony diameter [6].

### 2.2 Antagonistic interaction assay

Antagonistic interaction assay between the selected bacterial strains was characterized by the pour-plate method as described by Grossart et al. [25]. A molten nutrient 1% agar (2.5 mL) was mixed with 50 μL target bacterial strain suspension (1×10^8^ CFU/mL). The cell suspension agar was poured onto a nutrient agar plate (Sigma) and left for 10 min until it was completely solidified. An aliquot (10 μL) of test strain (1×10^8^ CFU/mL) was spotted onto the lawn and thereafter incubated at 30°C for 3 days. The strains were tested in triplicate against each other and observed daily for inhibition zones. Inhibitory activity was considered positive when the inhibition zone was more than 4 mm of the diameter of the spotted colony.

### 2.3 Determination of quantitative P and K solubilization in liquid culture

Another experiment was set up to estimate the quantitative P and K solubilization of the selected bacterial strains in liquid media. Bacterial cultures (inoculum adjusted to 1×10^8^ CFU/mL) were transferred to NBRIP and Aleksandrov liquid media separately. For bacterial synergistic interaction test, a mixed bacterial culture was prepared by mixing inoculum culture at equal ratio 1:1:1 v/v of strain 15a1, 38 and 40a before inoculation into test media. Cultures were grown at 30°C for 5 days on a rotary shaker at 150 rpm. The pH change of the liquid media was measured at the end of incubation time and cultures were centrifuged at 10,000 rpm for 10 min to obtain cell-free supernatant. The P solubilization in liquid media was determined by the yellow phospho-molybdo-vanadate colorimetry [26] using a UV-VIS spectrophotometer (Secomam Uviline 9400, France). The K solubilization was determined using an atomic absorption spectrometer (AAS) with the flame air-C_2_H_2_ at the wavelength of 766.5 nm [1,27].

### 2.4 Organic acid determination in post-incubation liquid culture

The cell-free supernatant of NBRIP and Aleksandrov liquid culture was filtered using a 0.22 μm nylon filter (Millipore, USA). About 20 μL of cell-free supernatant was passed through Rezex (Phenomenex) organic acid (ROA) column in Agilent high-performance liquid chromatography (HPLC) system at 60°C with a flow rate of 0.6 mL/min. Organic acid standard solutions were prepared beforehand which were citric, gluconic, acetic, GA3, malic, succinic and lactic acid at 10 mg/mL concentration. Quantitative estimation of organic acids was performed based on the comparison of peak area and retention time of sample with those of standards.

### 2.5 Bacterial growth curve, biochemical tests and molecular identification

The selected bacteria were identified by phenotypic and genotypic methods. The growth curve pattern of bacteria was determined using nutrient broth (Sigma) which received a standardized amount of 1 mL starting inoculum (1 × 10^8^ CFU/mL) in 250 mL Erlenmeyer flask. The culture was incubated at 37°C on a rotary shaker at 150 rpm and thereafter sampled (2 mL) every 2 h. At the time of sampling, absorbance reading at 600 nm was recorded using a UV/VIS spectrophotometer. In another experiment, bacteria were screened for a series of biochemical reaction tests: amylase [28]; [29]; protease [30]; lipase [31]; catalase; urease; acetate utilization. A light microscope (Olympus CH, Japan) was used to observe morphological characteristics and Gram’s reaction of the bacterial strains. Bacterial 16S rRNA gene was analyzed by employing DNA barcoding service at Apical Scientific Laboratory, Selangor, Malaysia, using BigDye® Terminator v3.1 cycle sequencing kit and sequenced by Applied Biosystems genetic analyzer platform.

The generated sequences were viewed and trimmed using BioEdit version 7 [32] and compared with the closest strains in the GenBank database using Basic Local Alignment Search Tool (BLAST) in terms of percent identity, query coverage and E-value. The sequences were aligned by ClustalW and the phylogenetic tree was reconstructed using MEGA X Alignment Explorer [33,34] and inferred by using the Neighbour-Joining method [35] with 1000 replicates of bootstrap values [36]. The evolutionary distances among strains were computed using p-distance [37]. Sequences were deposited into National Center for Biotechnology Information (NCBI) GenBank database via online sequence submission tool BankIt to retrieve the accession numbers.

### 2.6 Plant growth-promoting activity assays

The *Enterobacter* spp. were subjected to further screening for PGP activities e.g., N_2_ fixation, IAA production, GA3 production, zinc solubilization, siderophore and EPS production. The quantitative estimation of nitrogen fixation activity was performed using a modified micro Kjeldahl method [38]. Cultures were grown in N-free malate semisolid medium (NFb) [39] at 30°C for 10 days on a rotary shaker at 150 rpm. The change of colour of the light green NFb to blue indicates the ability of the bacterial strains to convert atmospheric N_2_ into ammonia. The percentage of fixed nitrogen was measured using sulfur digestion and distillation with NaOH [40]. About 2 mL of sample was digested with 2 mL concentrated H_2_SO_4_, 1 mL of 30% H_2_O_2_ and 0.7 g digestion mixture (100 g Na_2_SO_4_ + 10 g CuSO_4_.5H_2_O + 1 g selenium) at 350–375°C. The product of digestion was distilled with 10 mL NaOH 10 mol/L, and the ammonia trapped in 10 mL of 2% boric acid was measured by titration with H_2_SO_4_ 0.0025 mol/L.

Tryptic soy broth was employed to evaluate the IAA production of *Enterobacter* spp. with the addition of 0.1 g/L L-Tryptophan as the precursor of IAA in the culture composition [41]. Cell-free supernatant was mixed with 2 mL Salkowsky reagent containing 2% of 0.5 M FeCl_3_ in 35% perchloric acid [42] and was allowed to settle for 25 min. The intensity of pink colour was determined using a UV spectrophotometer at 535 nm and compared against the standard curve of pure IAA of known concentration.

The *Enterobacter* spp. were screened for zinc solubilization by using tris-minimal salt media which contains (per Liter): 10 g D-glucose, 6.06 g Tris-HCl, 4.68 g NaCl, 1.49 g KCl, 1.07 g NH_4_Cl, 0.43 g Na_2_SO_4_, 0.2 g MgCl_2_.2H_2_O, 0.03 g CaCl_2_.2H_2_O, and 15 g agar. The ZnO (1 g/L) was added in the media as the sole source of zinc to check the ability of the bacterial strains to solubilize zinc oxide [43]. The plate was incubated at 30°C for 5 days in triplicate and the formation of halo zone around colonies indicated zinc solubilization activity. Zinc solubilization index (ZSI) was calculated as the ratio of the total halo diameter to the colony diameter [6].

The siderophore production of *Enterobacter* spp. was assessed qualitatively as described by Lakhshmanan et al. [44] using chrome azurol S (CAS). The reagent was prepared by dissolving 0.0605 g CAS in 50 mL water and mixed with 10 mL FeCl_3_ solution (1 mM FeCl_3_.6H_2_0, 10 mM HCl). With constant stirring, the CAS-FeCl_3_ solution was slowly added into hexadecyltrimethylammonium solution (0.0729 g in 40 mL distilled water). The final mixture of 100 mL was added into 900 mL of autoclaved Luria broth (LB) agar, pH 6.8. Bacterial strains exhibiting a yellowish-orange halo zone after 10 days of incubation were considered as siderophore-producing strains.

EPS production of *Enterobacter* spp. was evaluated based on the formation of a mucoid colony on the agar plate-containing glucose such as the NBRIP and Aleksandrov agar after ten days of incubation. This biopolymer formation was verified by mixing a portion of the mucoid substance in 2 mL of absolute alcohol (99%). The formation of precipitate at the bottom of the solution indicated EPS production [45].

### 2.7 Biosafety inspection of *Enterobacter* spp.

Biosafety inspection of *Enterobacter* spp. was carried out using a blood agar medium to evaluate the haemolytic potential [46]. An overnight culture of *Enterobacter* spp. was streaked onto a blood agar plate comprising 5% (v/v) sheep blood and incubated at 30°C for 48 h. Haemolysis of red blood cells was assessed by the formation of a clear zone around colonies (*β*-haemolysis), or greenish colouration (*α*-haemolysis), while no clear zone indicates **γ**-haemolysis.

### 2.8 Okra seed biopriming and germination bioassay

Okra variety seeds of F_1_ Hybrid Okra-Best Five 304 (Green World) were surface sterilized with 70% ethanol and diluted Clorox® solution (3%), followed by triple washes with sterile distilled water. The sterilized seeds were dried in laminar flow with constant airflow at room temperature for 1 h and thereafter soaked in a freshly grown *Enterobacter* spp. culture (adjusted to 1×10^8^ CFU/mL) suspended in 0.85% phosphate-buffered saline (PBS) separately for 1 h. Three seeds were placed into every plant tissue culture tube containing 50 mL of 0.25% water agar [6] with the additional nutrient of Murashige and Skoog basal medium (4.4 g/L). The tubes were placed in a sterile tissue culture room at 26±2°C for 7 days under controlled light conditions. During the first 3 days (water imbibition period), the tubes were set in darkness, while for the next 4 days (radicle protrusion period), the tubes were set for 16-h light and 8-h darkness condition. The uninoculated seeds soaked in sterile PBS were used as control. The experiment was set up using a completely randomized design (CRD) with six replicates for each treatment where each tube contained three seeds.

Several parameters were examined after 7 days post-inoculation (DPI) such as the length of hypocotyl and radicle, and the number of lateral roots formed. Measurement was taken from six seedlings sampled randomly from each treatment. Germination in seeds was achieved when radicals are half the size of the seed. The percentage of germination and seedling vigor index were determined using the formula described by Anupama et al. [47]. The experiment was repeated twice.

Root colonization potential of *Enterobacter* spp. on the root tips of the okra seedlings was observed via electron microscopy. Root samples (1 cm) were prepared aseptically and placed into vials for each treatment. Glutaraldehyde (4%) was added into the vials to fix samples for 2 days at 4°C. Samples were washed three times with 0.1 M sodium cacodylate buffer of 30 min soaking each. Osmium tetroxide (1%) was used for the post-fixation of roots for 2 h at 4°C prior to rewashing three times with 0.1 M sodium cacodylate buffer. Roots were dehydrated with a series of acetone washing of different concentration from 35% to 100% at almost 1 h incubation time each. Critical point dryer Autosamdri®-815 was used for final dehydration for about 1 h before mounting onto the stub. Dehydrated roots were coated with gold using a sputter coater and transferred to slide for viewing using scanning electron microscopy (SEM) Jeol JSM-6400.

### 2.9 *In planta* evaluation of seed biopriming with *Enterobacter* spp.

P and K solubilizing *Enterobacter* spp. were evaluated for their PGP potential by pot experiment using Holland peat moss under net house conditions. The experiment was performed at Research Laboratory 1.6, Bioprocessing and Biomanufacturing Research Center, Universiti Putra Malaysia (3°00'08.3"N 101°42'12.0"E) during the rainy season (November–December 2019). Okra variety seeds of F_1_ Hybrid Okra-Best Five 304 (Green World) were surface sterilized and bioprimed with *Enterobacter* spp. as described previously, except for uninoculated seeds where sterile PBS was used for priming. Carrier material of autoclaved peat moss (50 g per pot) was mixed with an overnight culture of *Enterobacter* spp. (1×10^8^ CFU/mL) and left for 1 h.

Inoculated seeds were sown in sowing pots with 8 cm diameter containing 0.2 Kg peat moss which was previously mixed with the prepared carrier. Pots were watered thoroughly after sowing to serve a good moisture condition for a favourable seed germination. There were three seeds per pot with six replicates under four different treatments and arranged in CRD. Seedlings were uprooted after 25 days of sowing and studied for plant growth parameters e.g., shoot length, root length, wet and dry biomass. Leaf surface area was measured using calculation described by Alami et al. [48] while the chlorophyll index was determined using Chlorophyll Meter SPAD-502 Plus (Konica Minolta) according to the standard manual. The experiment was repeated twice successively. Soil viable cell was quantified by a standard serial dilution method using PBS (pH 6.8) and nutrient agar.

### 2.10 P and K content determination of seedlings and soil

The peat soil, leaf, stem and roots of the uprooted seedlings were dried at 70°C for 48 h until a constant weight was achieved. Samples were ground into a fine powder and subjected to one-step concentrated nitric acid digestion [49] for total P and K content estimation. Available P and K were determined by sodium carbonate extraction method [50]. The determination of P and K content was carried out by inductively coupled plasma optical emission spectrophotometer (ICP-OES) Optima 7300DV (Perkin Elmer) at 213.617 nm (P analyte) and 766.49 nm (K analyte).

### 2.11 Statistical analysis

Mean comparison of solubilization index, pH of culture media, soluble P and K, germination, seedling growth parameters and the P and K content were analyzed using one-way analysis of variance (ANOVA) by using SPSS software Package Version 25.0 (SPSS Inc., USA). The difference between treatments was compared by the least significant difference (LSD) test at P<0.05.

## 3 Results

### 3.1 Preliminary screening of P and K solubilizing bacteria

All bacterial cultures were retrieved from glycerol stocks of our previous bacterial isolation works from several sources, such as from agricultural soil, oil palm empty fruit bunch (OPEFB) and chicken manure co-composting process. A total of 18 phenotypically different bacterial strains were subcultured and strictly selected from those which were able to optimally solubilize inorganic P and K on solid NBRIP and Aleksandrov agar. Three out of eighteen bacterial strains, labelled as 15a1, 38 and 40a (isolated from OPEFB-manure compost) exhibited the largest halo zone on agar media for both P and K solubilization activity (Table 1). The PSI and KSI of the three bacterial strains were determined in the next experiment.

**Table 1:** Qualitative P and K solubilization screening of *Enterobacter* spp.

Strain ID	P solubilization	K solubilization
15a1	++	++
38	++	++
40a	++	++
1c	–	–
Sp4	–	–
2b	–	–
2d55	–	–
10c	–	–
12a	–	–
14a	–	–
15–1	–	–
15a2	++	–
16b35	–	–
20b	+	–
22a	–	–
Sb16	+	–
Psb16	+	–
Bc	+	–

P and K solubilization were categorized as low activity when the halo zone is < 5 mm.

(denoted as +) or high activity when the halo zone > 5 mm (denoted as ++).

**Fig 1:**
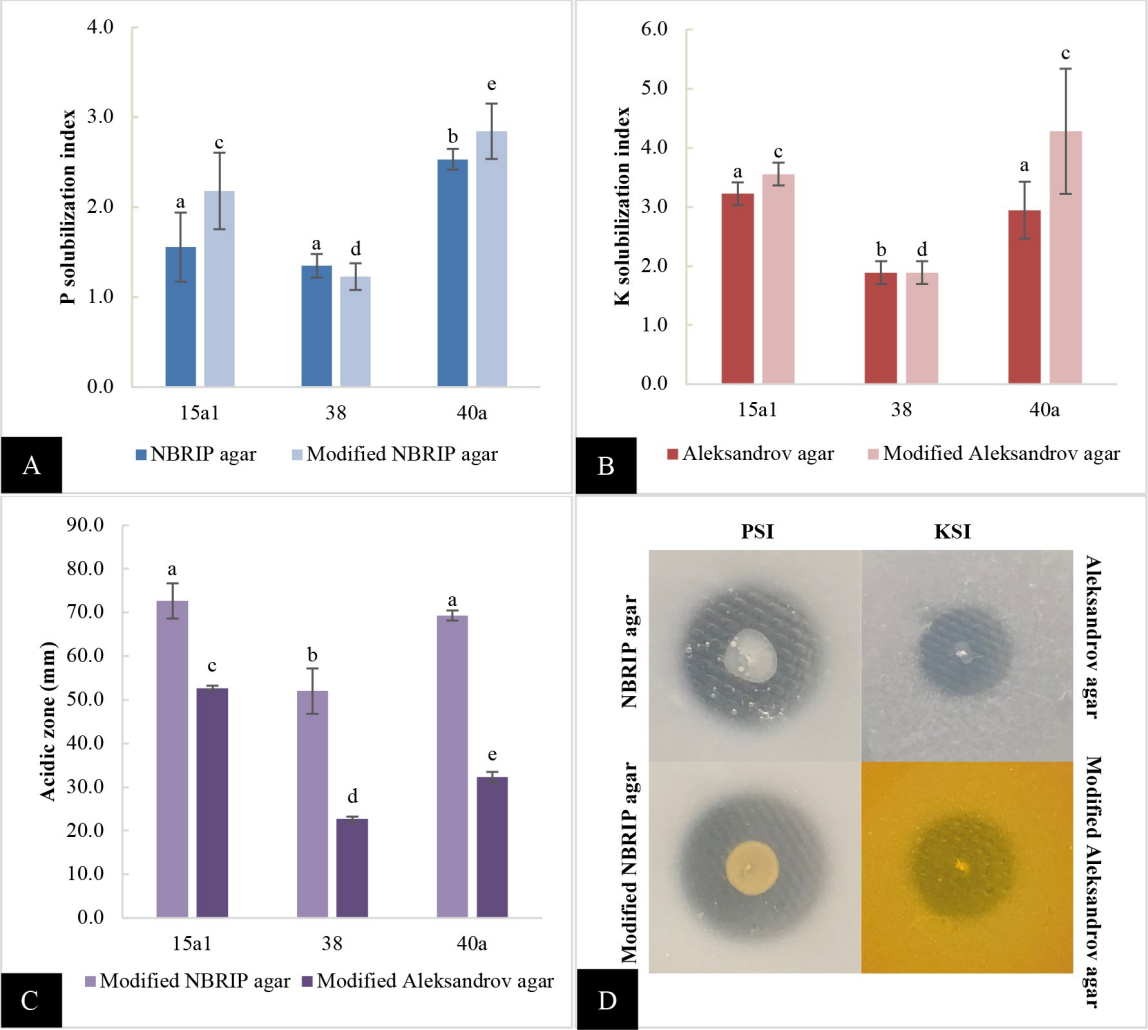
*In vitro* P and K solubilization index of *Enterobacter* spp. on different solid agars. A: PSI on NBRIP and modified NBRIP agar, B: KSI on Aleksandrov and modified Aleksandrov agar, C: acidic zone on modified NBRIP agar and modified Aleksandrov agar, D: halo formation due to solubilization of insoluble P and K on different agars. All values are the mean value of three biological replicates alongside the standard deviation indicated by the error bar. Means followed by different letters in the same category indicate significant differences at P<0.05.

Based on *in vitro* plate screening (Fig 1), *E*. *hormaechei* 40a produced the highest PSI (2.5 on NBRIP, 2.8 on modified NBRIP) while *E*. *hormaechei* 15a1 surpassed *E*. *clocae* 38 and *E*. *hormaechei* 40a for KSI level (3.2 on Aleksandrov agar). However, *E*. *hormaechei* 40a showed the highest KSI (4.3) on modified Aleksandrov agar as compared to the other two strains. All of them showed a prominent size of the acidic zone on both modified NBRIP and Aleksandrov agar containing pH indicator dye with *E*. *hormaechei* 15a1 and 40a showing the largest zone on modified NBRIP). However, the PSI and KSI of both standard and modified media were not significantly different (P>0.05).

A mixed culture of *Enterobacter* spp. was prepared to contain an equal ratio of inoculum size as stated in the previous section. Based on plate assay, there were no observable inhibitory activities that occurred during the growth of bacteria against each other. It was presumed that the mixed culture could demonstrate synergistic activities when tested for the subsequent PGP trait assays. Thus, the following qualitative estimation of P and K solubilizing activities of *Enterobacter* spp. incorporated both individually and mixed culture experiments.

### 3.2 Quantitative estimation of P and K solubilizing activity and organic acid production

**Fig 2:**
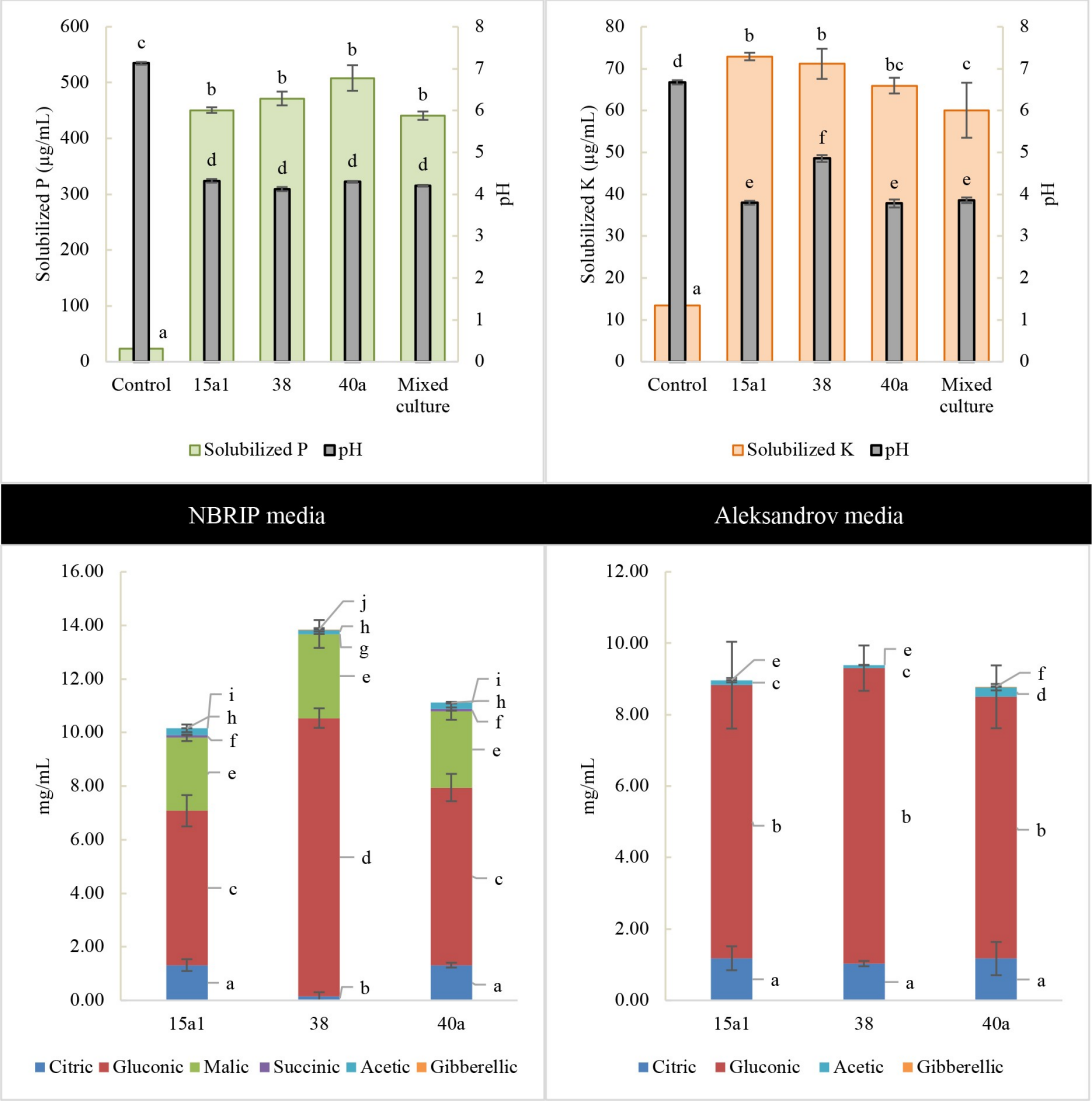
Interaction between pH level and solubilization of P and K in liquid NBRIP and Aleksandrov media respectively after 4^th^ of day of incubation. Organic acids produced by *Enterobacter* spp. were quantified using Agilent HPLC. All values are the mean value of three biological replicates alongside the standard deviation indicated by the error bar. Means followed by different letters in the same category indicate significant differences at P<0.05.

In the quantitative assay of P solubilizing activity, *E*. *hormaechei* 40a consistently demonstrated the highest soluble P (508.25 μg/mL) followed by *E*. *clocae* 38 (471.58 μg/mL) and *E*. *hormaechei* 15a1 (450.75 μg/mL) in NBRIP media after 5-day incubation (Fig 2). Contrary to K solubilizing activity, *E*. *hormaechei* 15a1 released the highest soluble K (72.90 μg/mL) followed by *E*. *clocae* 38 (71.15 μg/mL) and *E*. *hormaechei* 40a (65.95 μg/mL) in Aleksandrov culture. The pH level of the inoculated cultures was significantly higher (P<0.05) than uninoculated control media and ranged between acidic pH of 3.78 (40a in Aleksandrov broth) to 4.85 (38 in Aleksandrov broth). However, the mixed culture did not show any synergistic effects on the solubilization of P and K as well as the pH level of the culture in comparison with those individual cultures (P>0.05).

The production of organic acids by *Enterobacter* spp. was identified using HPLC and the resulting chromatogram profiles were analysed against the standards (S2 Fig). In NBRIP culture, all three bacteria produced a prominent amount of gluconic acid, malic acid and citric acid. *E*. *clocae* 38 produced the highest amount of gluconic acid (10.39 mg/mL) followed by malic acid (3.14 mg/mL) while both *E*. *hormaechei* 15a1 and 40a produced higher amount of citric acid (1.32 mg/mL) as compared to *E*. *clocae* 38. Succinic, acetic and GA3 acid were produced in a small amount (< 0.3 mg/mL) by *E*. *hormaechei* 15a1 and 40a. Likewise in Aleksandrov media, all *Enterobacter* spp. produced a prominent amount of gluconic and citric acid. Contrary to NBRIP media, a minimal amount (< 0.3 mg/mL) of acetic acid was detected in Aleksandrov media, while a consistent low amount of GA3 acid was detected in both media.

### 3.3 Phenotypic and molecular identification of bacterial strains

The growth curve of *Enterobacter* spp. was recorded by optical density (600 nm) using nutrient broth throughout 24-h incubation (S1 Fig). The maximum cell density recorded for *E*. *hormaechei* 15a1 was at 18^th^ h while the cell growth peak of *E*. *clocae* 38 and *E*. *hormaechei* 40a was recorded at 20^th^ h. Cell morphology observation under a light microscope revealed that cells were single coccobacillus and Gram-negative. The bacterial colony was observed as round, white, entire, raised, butyrous and was around 0.8 to 1.5 mm diameter size on nutrient agar.

Biochemical characterization assay of *Enterobacter* spp. showed that none of the strains exhibited hydrolytic activities such as amylase, cellulase, protease and lipase activities (Table 2). Catalase and urease assay were tested positive for all *Enterobacter* spp. while acetate utilization was solely demonstrated by *E*. *cloacae* 38. PCR amplification of 16S rRNA gene sequencing was employed to identify the P and K solubilizing strains. Sequences were compared on the NCBI BLASTN tool based on percentage similarity, E-value and query coverage (S1 Table). BLAST result of 15a1 and 40a presented 100% similarity with *Enterobacter hormaechei* whereas 38 showed 100% similarity with *Enterobacter cloacae*. Sequences were deposited to the NCBI GenBank database and the unique accession numbers were retrieved (MN294583.1, MN294584.1 and MN294585.1).

**Table 2:** Cell and colony morphology, biochemical characteristics and molecular identification of *Enterobacter* spp.

Characteristics	Strain		
	15a1	38	40a
Cell morphology	Single coccobacillus, Gram-negative		
Colony morphology	Round, white, entire, raised, butyrous, 0.8–1.5 mm		
Amylase production	–	–	–
Cellulase production	–	–	–
Protease production	–	–	–
Lipase production	–	–	–
Catalase production	+	+	+
Urease production	+	+	+
Acetate utilization	–	+	–
Closest relatives in NCBI GenBank	*Enterobacter hormaechei* strain CPO 4.200 (MN733028.1)	*Enterobacter cloacae* strain BAB-2824 (KF535159.1)	*Enterobacter hormaechei* strain CPO 4.200 (MN733028.1)
Percentage similarity	100.00%	100.00%	100.00%
Designated species name	*Enterobacter hormaechei* 15a1	*Enterobacter cloacae* 38	*Enterobacter hormaechei* 40a
NCBI Accession no.	MN294583.1	MN294584.1	MN294585.1

+ and–symbols indicate positive and negative reactions respectively.

**Fig 3:**
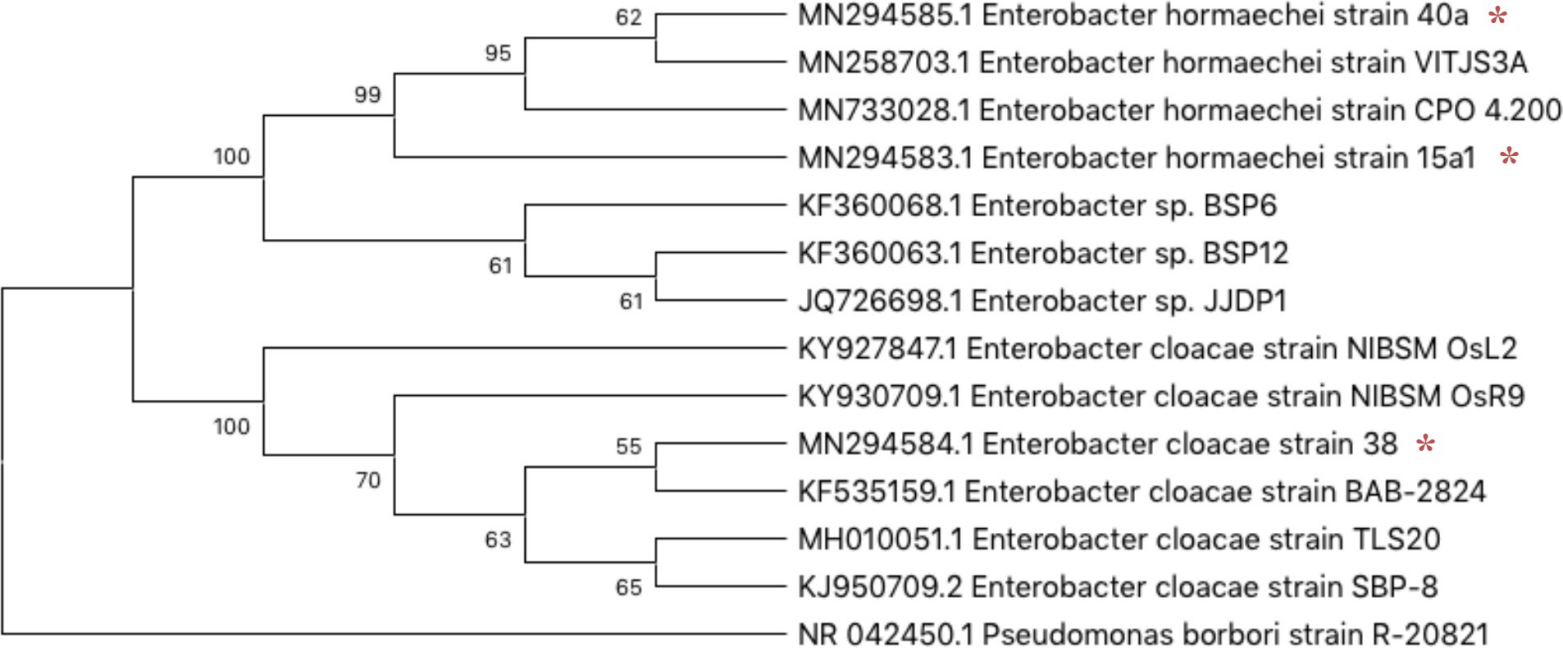
Molecular identification of *Enterobacter* spp. via 16S rRNA gene sequencing. Optimal phylogenetic tree of the bacterial strains (indicated with asterisk) and their closest strains was reconstructed by the Neighbor-joining method with the sum of a branch length of 0.14724404 using MEGA X Alignment Explorer.

The evolutionary relationship was reconstructed using the neighbour-joining method to generate the optimal phylogenetic tree with the sum of branch length of 0.14724404 (Fig 3). The numbers shown next to the branches represented the replicate trees percentage in which the related taxa were grouped together in the bootstrap test of 1000 replicates. p-distance was used to compute the evolutionary distances among strains which in total involved 14 nucleotide sequences. Pairwise deletion was opted to eradicate ambiguous positions for each sequence pair. The final dataset consisted of 1506 positions and all evolutionary analyses were performed using MEGA X Alignment Explorer.

### 3.4 Multi-putative plant growth-promoting activity of *Enterobacter* spp.

*Enterobacter* spp. were also examined on the ability to demonstrate multi-putative PGP activities such as N_2_ fixation, IAA production, GA3 production, zinc solubilization, siderophore and exopolysaccharide (EPS) production (Table 3). N_2_ fixation of *Enterobacter* spp. was detected by the colour change of the light green NFb to blue indicating ammonia production from the conversion of atmospheric N_2_. It was later confirmed by the micro Kjeldahl method that all strains were able to fix N_2_ whereby *E*. *cloacae* 38 fixed the highest N_2_ (2.10 μg N/mL) followed by *E*. *hormaechei* 15a1 (1.23 μg N/mL) and *E*. *hormaechei* 40a (0.36 μg N/mL).

**Table 3:** Multi-putative plant growth-promoting characteristics of *Enterobacter* spp.

Strain ID	Fixed N_2_ (μg N/mL)	IAA (μg/mL)	GA3 (μg/mL)	Zinc	Siderophore	EPS
*E*. *hormaechei* 15a1	1.23±0.51	110.81±0.83	NA	–	++	–
*E*. *cloacae* 38	2.10±0.82	184.03±0.93	21.05±0.51^a^	–	+	+
*E*. *hormaechei* 40a	0.36±0.44	57.51±2.81	23.16±0.91^b^	–	++	–

Gibberellic acid was detected using HPLC system in NBRIP

^a^ or/ and Aleksandrov

^b^ culture

Data are the mean value of three biological replicates with ± denoting standard deviation. + and–indicates positive and negative traits respectively while ++ indicates high intensity qualitatively.

Additionally, *E*. *cloacae* 38 produced the highest IAA in LB-tryptophan media compared to the other two strains. While in GA3 assay, both *E*. *cloacae* 38 and *E*. *hormaechei* 40a produced a detectable amount of GA3 while none was detected for *E*. *hormaechei* 15a1. Zinc solubilization was not detected for all strains since no halo zone was exhibited surrounding the bacterial colonies. Siderophore production was tested positive for all strains when intense yellowish-orange halo zone appeared on plates around bacterial colonies after the 5^th^ day of incubation. However, only *E*. *cloacae* 38 was observed to produce EPS on both NBRIP and Aleksandrov agar plates with the formation of mucoid-like colonies (S2 Fig).

### 3.5 Biosafety characteristic of *Enterobacter* spp.

Blood agar was used to identify the biosafety characteristic of the *Enterobacter* spp. Haemolytic activity was not exhibited by all strains since no detectable zone of clearings (**γ**-haemolysis) by bacterial colonies on the blood agar (S2 Fig). This could indicate that the *Enterobacter* spp. may not be pathogenic for humans, thus safe for further plant bioassay studies.

### 3.6 Germination assay and colonization detection of *Enterobacter* spp.

**Fig 4:**
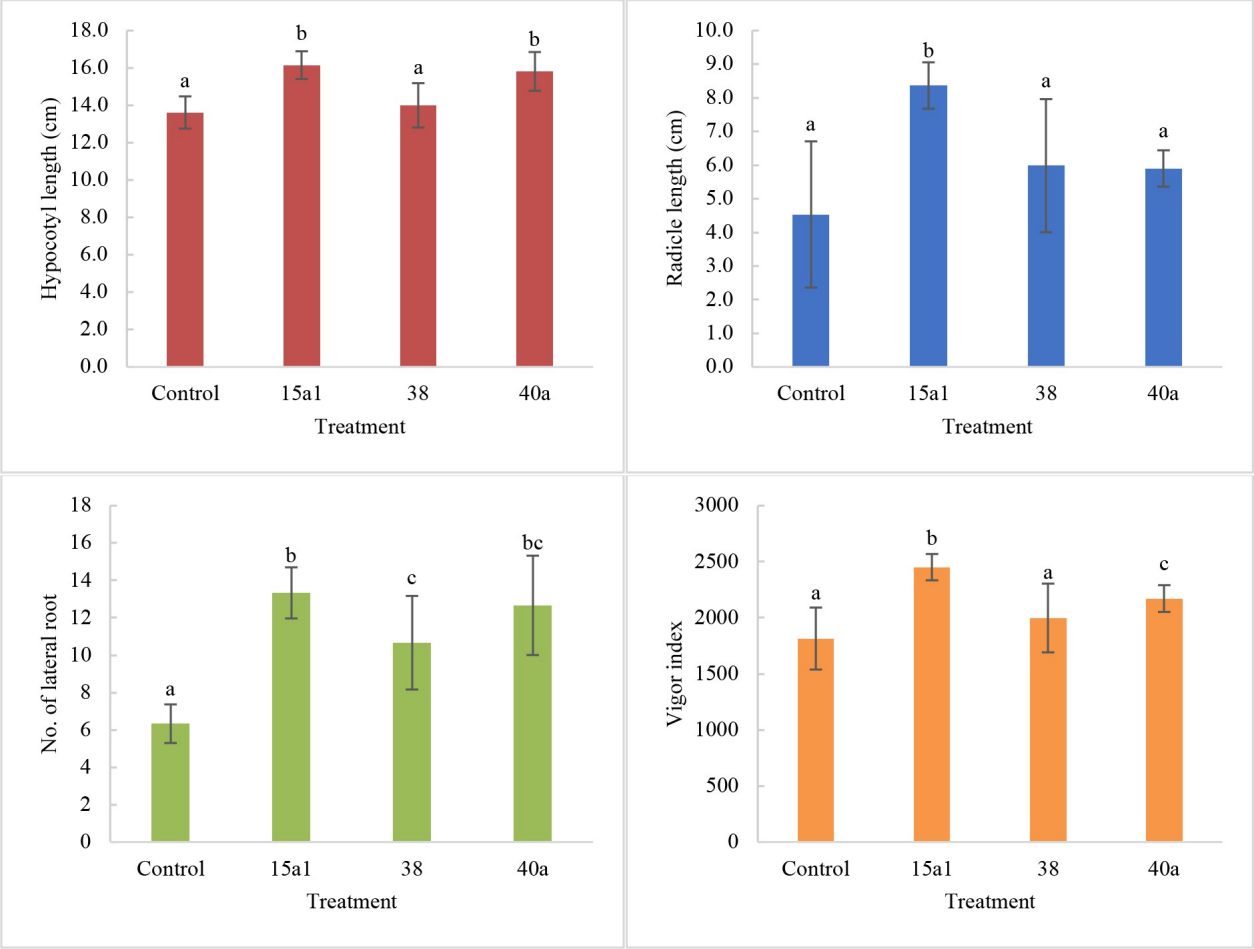
Effects of seed biopriming on germination of okra seeds in a culture tube assay under sterile tissue culture room conditions. All values are the mean value of six biological replicates alongside the standard deviation indicated by the error bar. Means followed by different letters in the same graph indicate significant differences at P<0.05.

Germination bioassay was carried out to evaluate the effects of *Enterobacter* spp. on the early vegetative growth stage of okra seedling. Seed biopriming using the *Enterobacter* spp. culture significantly improved (P<0.05) okra early seedling growth as compared to the uninoculated control seeds based on the length of hypocotyl and radicle, number of lateral roots and vigor index (Fig 4). The longest hypocotyl (16.2 cm) and radicle length (8.4 cm) as well as the highest number of lateral roots (13.3) were recorded by seeds bioprimed with *E*. *hormaechei* 15a1. Although the germination percentage remained as 100% for all treatment, the vigor index showed significant improvement for bioprimed seeds where the highest index was recorded by seeds treated with *E*. *hormaechei* 15a1 (2451.7) followed by *E*. *hormaechei* 40a (2171.7) and *E*. *cloacae* 38 (1998.3) against the untreated control seeds (1815).

**Fig 5:**
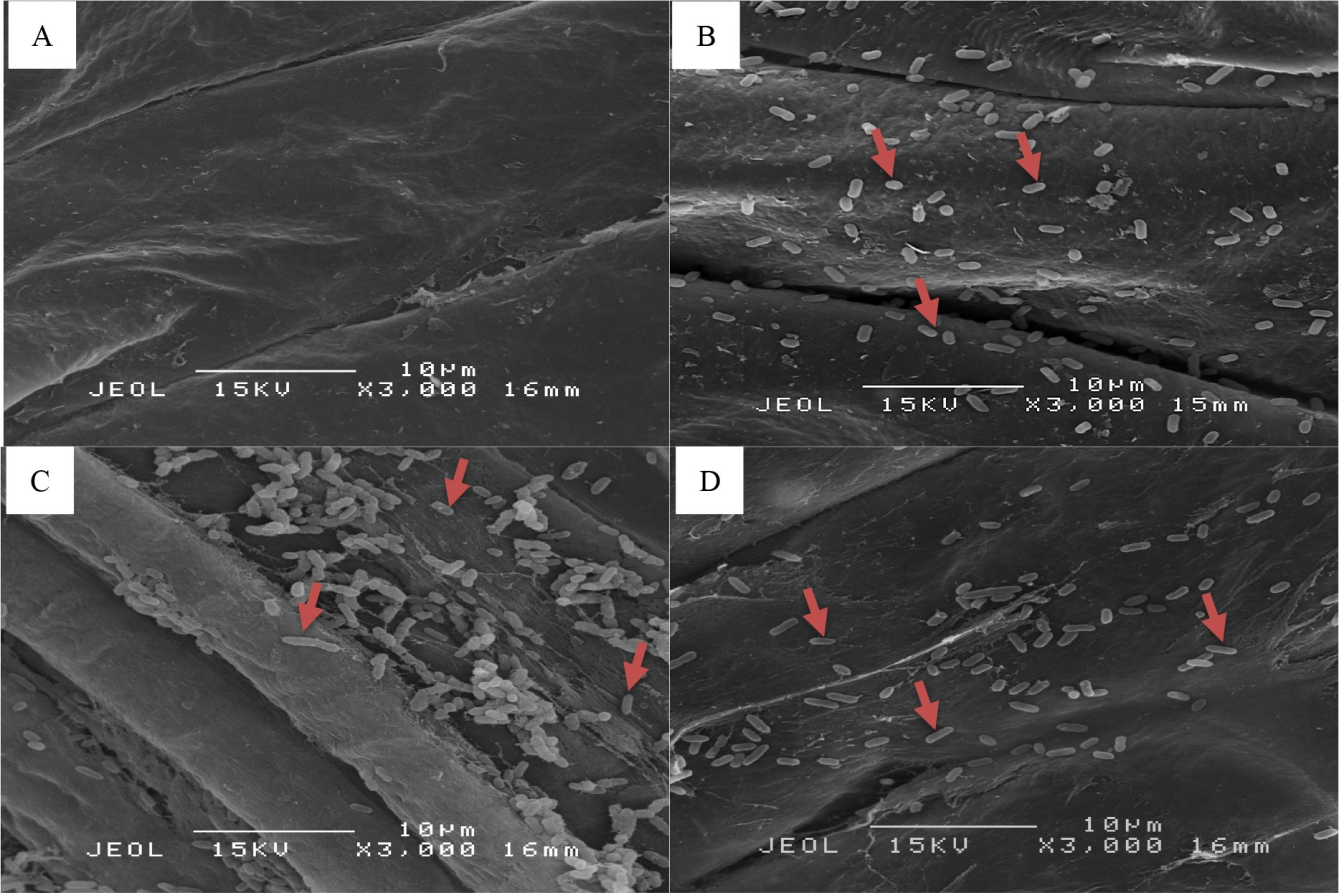
Images of root sections indicating *in situ* colonization of bacteria (indicated by arrow) under controlled germination assay viewed using SEM. A: Control (uninoculated), B, C and D: Root colonization of *E*. *hormaechei* 15a1, *E*. *cloacae* 38 and *E*. *hormaechei* 40a respectively.

To verify the effective colonization of *Enterobacter* spp. on okra seedling roots under sterile condition, the chemically fixated root samples were viewed using SEM. Images of root sections viewed under 3000× magnification (Fig 5) revealed that the *Enterobacter* spp. successfully colonized root surface of okra seedlings where the total bacterial population for roots colonized by *E*. *hormaechei* 15a1 was 0.19 cells/μm^2^, *E*. *cloacae* 38 was 0.45 cells/μm^2^ and *E*. *hormaechei* 40a was 0.25 cells/μm^2^.

### 3.7 Effects of seed biopriming with *Enterobacter* spp. on okra seedling growth

Since *Enterobacter* spp. seed biopriming gave out positive effects on okra seed vigor index, a seedling pot experiment was carried out to evaluate their PGP effects under net house conditions. After 25 days of post-biopriming, seedlings were uprooted and analysed as described in the previous section. *Enterobacter* spp. seed biopriming improved early vegetative growth of okra seedlings under net house conditions. Okra seedlings treated with *Enterobacter* spp. showed a significant increase (P<0.05) in overall shoot and root length as well as plant biomass (Table 4). The shoot length of the bioprimed seedlings ranged between 17 cm to 17.4 cm and the root length ranged between 21.4 cm to 24.1 cm. While the shoot length of the uninoculated seedlings was 14.8 cm and the root length was 18.8 cm. Seedlings treated with *E*. *hormaechei* 40a showed the highest fresh and dry biomass of both shoot and root plant parts.

**Table 4:** Effects of *Enterobacter* spp. on early vegetative growth of okra seedlings under net house conditions.

Treatment	Shoot			Root		
	Length (cm)	Fresh biomass (g)	Dry biomass (g)	Length (cm)	Fresh biomass (g)	Dry biomass (g)
Control (uninoculated)	14.8±1.8a	1.32±0.16a	0.24±0.03a	18.8±3.3a	0.88±0.37a	0.12±0.04a
*E*. *hormaechei* 15a1	17.4±0.6b	1.73±0.23b	0.28±0.04a	22.1±2.2b	1.76±0.41b	0.16±0.03ab
*E*. *cloacae* 38	17.0±1.2b	1.91±0.29b	0.28±0.06a	21.4±1.9ab	1.94±0.26b	0.18±0.04b
*E*. *hormaechei* 40a	17.0±0.8b	1.97±0.15b	0.28±0.03a	24.1±1.5b	2.03±0.24b	0.18±0.03b

Data are the mean value of six biological replicates with ± symbol denoting standard deviation. Means followed by different letters in the same column indicate significant differences at P<0.05.

Leaf surface area and SPAD chlorophyll index of bioprimed seedlings were increased significantly (P<0.05) as compared to uninoculated seedlings (Table 5). Treatment using *E*. *hormaechei* 40a showed the highest improvement in leaf surface area (18.5 cm^2^) followed by *E*. *hormaechei* 15a1 (18.5 cm^2^) and *E*. *cloacae* 38 (15.7 cm^2^). Likewise, the SPAD chlorophyll index of the leaves of the bioprimed seedlings was increased up to 37.9. Soil viable cell of bioprimed seedlings recorded a higher CFU ranged from 6.0 × 10^7^ to 9.5 × 10^7^ CFU/g soil as compared to uninoculated seedlings (3.5 × 10^7^).

**Table 5:** Effects of *Enterobacter* spp. on leaf surface area, SPAD chlorophyll index and soil viable cell.

Treatment	Leaf surface area (cm^2^)	SPAD Chlorophyll index	Soil viable cell (CFU/g)
Control (uninoculated)	13.2±1.7a	34.6±1.9a	3.5 × 10^7^
*E*. *hormaechei* 15a1	18.5±2.1b	36.6±1.9ab	8.0 × 10^7^
*E*. *cloacae* 38	15.7±1.5c	37.9±2.0b	6.0 × 10^7^
*E*. *hormaechei* 40a	18.8±1.2b	37.9±1.7b	9.5 × 10^7^

Data are the mean value of six biological replicates with ± symbol denoting standard deviation. Means followed by different letters in the same column indicate significant differences at P<0.05.

### 3.8 Effects of *Enterobacter* spp. on P and K uptake

The original P and K content in soil were analysed prior to the pot experiment as follows: pH 7.5; total P 0.65 mg/g; available P 36.4 μg/g; total K 1.56 mg/g; available K 57.6 μg/g. The P and K contents in plants were evaluated separately by plant sections which were the leaf, stem and root along with the medium soil. The P and K uptakes of the bioprimed seedlings were significantly higher (P<0.05) as compared to the uninoculated seedlings (Table 6). The range of the total P content in plant sections of the bioprimed seedlings was around 1.54 to 3.54 mg/g while the uninoculated seedlings was around 1.54 to 2.54 mg/g only. The seedlings bioprimed with *E*. *hormaechei* 40a consistently showed the highest total P content in all plant parts including the leaf, stem, root and soil. The total K content of the leaf, stem and root of the bioprimed seedlings were observed higher than the uninoculated seedlings. The soil available P and K were increased for all bioprimed seedlings as compared to the uninoculated seedlings.

**Table 6:** Effects of *Enterobacter* spp. on P and K uptake of okra seedlings and soil available P and K under net house conditions.

Treatment	Leaf	Stem	Root	Soil	Soil available P content (μg/g)
	Total P content (mg/g)				
Control (uninoculated)	2.05±0.02a	2.51±0.07a	1.54±0.02a	0.25±0.01a	17.20±2.80a
*E*. *hormaechei* 15a1	2.90±0.05b	3.18±0.07b	1.60±0.02b	0.28±0.01b	25.47±0.92b
*E*. *cloacae* 38	2.63±0.09c	2.54±0.03a	1.54±0.03a	0.27±0.01b	24.53±2.05b
*E*. *hormaechei* 40a	3.01±0.01d	3.54±0.14c	1.91±0.02b	0.32±0.01c	26.27±2.20b
Treatment	Total K content (mg/g)				Soil available K content (μg/g)
Control (uninoculated)	9.79±0.08a	11.00±0.18a	12.29±0.08a	0.51±0.02a	5.20±2.23a
*E*. *hormaechei* 15a1	16.10±0.12b	15.37±0.67b	16.85±0.55b	0.64±0.02b	9.20±2.00b
*E*. *cloacae* 38	15.67±0.61b	13.89±0.45c	13.94±0.35c	0.53±0.02a	6.93±0.23a
*E*. *hormaechei* 40a	18.49±0.49c	17.83±0.35d	17.99±0.14d	0.75±0.02c	12.00±1.83b

Data are the mean value of six biological replicates with ± symbol denoting standard deviation. Means followed by different letters in the same column indicate significant differences at P<0.05.

## 4 Discussion

The exorbitant cost of chemical fertilizer and the limited sources of unsustainable P- and K-based mineral extraction have driven the search for alternative strategies to meet the agricultural demand for fertilizer input. The use of plant-beneficial microbes for agriculture to improve crop productivity has been taken seriously by a number of countries worldwide due to its notable efficacy. The present study was primarily designed to screen for P- and K- solubilizing bacteria and thereafter evaluated for other plant beneficial traits that were suitable for agricultural application. Besides, the root colonization ability of the strains was observed and inferred as a bacterial symbiotic mechanism to make way for available nutrients for plant nutrition. To this end, the pot experiment was applied to assess the efficiency of the strains to improve the early vegetative growth of okra seedlings via seed and soil biopriming technique.

Out of 18 phenotypically different strains, 8 strains exhibited halo zone on NBRIP agar while only 3 strains showed halo zone on Aleksandrov agar. A modified NBRIP and Aleksandrov agar were incorporated in the subsequent experiment using indicator dyes to observe the pH change of agar due to P and K solubilization activity [23,24]. The PSI and KSI of the modified agar were observed more sensitive than the standard agar, as they mostly showed a higher index than the standard agar. However, it was found that both PSI and KSI between the two types of media were not significantly different (P>0.05). The modified agar can be regarded as an alternative indicator for P and K solubilizing activity of bacteria since it can provide additional information for the qualitative interpretation of results which are the primary PSI and/ or KSI data, as well as the pH change zone, which are the preliminary sign of active organic acid production.

The selected strains with both P and K solubilizing ability were subjected to antagonistic interaction assay where the strains were grown against each other. None of the strains demonstrated inhibitory activity, thus presumptively suitable for mixed culture experiments in quantitative estimation of P and K solubilization test. However, in the quantitative P and K solubilization assay, the mixed culture neither show synergistic effects of solubilization nor the reduction of pH of culture (P>0.05). This phenomenon could be due to the similar biological factors of those strains since they were closely related to each other and even a similar genus. In contrast to the previous report, the co-culture of distinctive species between *Burkholderia* spp. and *Aspergillus* spp. was able to demonstrate a significantly synergistic P solubilizing capacity [51]. Therefore, it is deduced that a synergistic effect is rather plausible when a strain with a desired specific activity is paired with another strain with distinctive beneficial traits as to complement each other to serve a collective purpose [1].

Previous studies have reported that there is a direct connection between the bacterial pathway for P solubilization and the secretion of multiple cellular organic acids [6]. In this study, the organic acid production by *Enterobacter* spp. was analysed using HPLC whereby a notable amount of gluconic acid was detected, followed by malic, citric, succinic, acetic and GA3 acid. The intense amount of gluconic acid presented in the growth media was a sign of the active conversion from its glucose precursor via the extracellular oxidation mechanism during the P solubilization [52]. This could explain the acidic pH of NBRIP and Aleksandrov cultures during the growth of *Enterobacter* spp. which ranged from 3.78 to 4.85. We suspected that both P and K solubilization by *Enterobacter* spp. employed a similar or partially similar pathway of remobilization since both NBRIP and Aleksandrov culture contained a spiked level of gluconic acid as compared to the other organic acids produced. In particular, Gupta [53] stated that P solubilizing bacteria such as *Bacillus* spp. and *Pseudomonas* spp. commonly secrets organic acids which lower the soil pH and cause the dissociation of complex P making them available to plants. Meanwhile, Itelima et al., [54] reported that the organic acids produced by K solubilizing microorganisms cause the dissolution of K-silicates and helps in the elimination of metal ions thereby serving the soluble K to plants. However, further investigation is required to understand the action of mechanisms involved during the P and K solubilization by the *Enterobacter* spp.

PGP trait assays revealed that the *Enterobacter* spp. were able to fix N_2_, produce IAA, GA3, siderophore and EPS. Most PGPB in the previous reports regardless of their respective genera often demonstrated more than one PGP traits [6,55,56]. N_2_-fixing ability is an important trait as a potential plant bioinoculant since N_2_ is the top essential macro-nutrient required for plant growth. IAA lies in the cluster of auxins family produced by plants or certain microbes as it is important for leaf formation, embryo development and root elongation [57]. A recent study has identified that *Enterobacter* sp. was able to secret IAA up to 5561.7 μg/mL using an optimized culture condition [58]. However, in this report, none of the strains was able to show zinc solubilization activity. Zinc solubilization is commonly exhibited by rhizobacteria including those of *Enterobacter* sp., *Pseudomonas* sp. and *Bacillus* spp. and this trait has been linked to the growth promotion of various plant varieties [6,43].

The *Enterobacter* spp. were confirmed as non-haemolytic via blood haemolysis test and presumptively safe for humans and animals [46]. *Enterobacter* spp. in general, like some other well-explored bacterial species associated with PGP abilities such as *Bacillus cereus*, *Bacillus anthracis*, *Enterobacter cloacae*, *Enterobacter amnigenus* and *Klebsiella pneumoniae*, are regarded as opportunistic human pathogen [59]. However, the virulence factors and pathogenicity of a species were reported to vary across different strains of similar species based on the genetic differences of virulence-associated genes carried by the specific strain [13]. For instance, Liu et al. [13] have reported that the pathogenicity islands that encode a Type III secretion system (T3SS) or a Type IV secretion system (T4SS) were only detected in *Enterobacter cloacae subsp*. *cloacae* ATCC13047, and they were absent in the other three *E*. *cloacae* strains that were tested together. Besides, biosafety determination using the blood haemolysis test has been widely used by many studies related to the isolation of plant-beneficial bacteria from environmental sources such as soil and plant parts [6,60,61]. This method, although inconclusive, provides preliminary information on the suitability of bacterial strains for human handling. Further biosafety evaluation on the *Enterobacter* spp. is imperative prior to its field application and commercialization.

Under the *in vitro* germination assay, seed biopriming with *Enterobacter* spp. improved okra early seedling growth as compared to the uninoculated control seeds based on the length of hypocotyl and radicle, number of lateral roots and vigor index. Effective root colonization of *Enterobacter* spp. was observed using SEM where a large number of bacterial cells were detected on root tips. This finding was comparable to the *in vitro* plate bioassay carried out in the previous report [6] as the seed inoculation of wheat by P solubilizing bacteria (*Enterobacter* spp. MS32) increased the germination and seedling growth as well as the root volume and tips size. Similarly, seed biopriming with a series of endophytic bacteria on muskmelon seeds resulted in the promotion of hypocotyl and radicle growth [62].

In the pot experiment of okra seedling, *Enterobacter* spp. showed the potential to improve the growth of the plant as indicated by the overall shoot and root length as well as the plant biomass. Besides, the leaf surface area and the SPAD chlorophyll index of the bioprimed seedlings were increased significantly (P<0.05) as compared to the uninoculated seedlings. The increase of total P and K content in the bioprimed seedlings as compared to control plants could explain the reason behind the growth promotion of okra seedlings. The soluble P and K in the soil matrix were also increased providing more readily available nutrients for plant uptake. Since P and K availability is considered a limiting step in plant growth and nutrition [63], this evidence suggests a significant contribution of the P- and K-solubilizing *Enterobacter* spp. to promote the okra plant growth since the early vegetative stage of seedling. Additionally, a recent review of biopriming techniques validated that the major microbial contributions towards enhanced plant productivity included and not limited to, N_2_ fixation; P and K remobilization; iron sequestration; microbial phytohormones, vitamins, functional enzymes, and beneficial organic acids secretion; nutrient uptake facilitation via hyphal networks; active metabolites production; seed emergence promotion; vigorous seedlings establishment [64].

## 5 Conclusion

To the best of our knowledge, this is the first report to simultaneously focus on both P and K solubilization by *Enterobacter hormaechei* sp., highlight the plant beneficial traits and apply to pot experiment to measure the plant nutrient uptake on okra seedlings using seed biopriming technique. Specifically, the increase in the available P and K in the soil was observed which corresponded to the higher P and K uptakes of the leaf, stem, root and shoot of the okra seedlings. Therefore, the P and K solubilizing *Enterobacter* spp., especially the *Enterobacter hormaechei* 40a might be considered as a promising candidate for P and K solubilizing biofertilizer for plant growth promotion to enhance seed germination to the subsequent early vegetative growth of seedlings and eventually the yield components. Further studies on genomic and metabolomic facets of this species might be meaningful as to comprehend its broad potential in promoting crop productivity particularly the systems-level response to metabolic lesions in biochemical pathways of enzymes, their physiological and ecological roles that might be appreciated in agriculture.

## Supporting information

S1 Fig Growth of *Enterobacter* spp. in nutrient broth at 37°C shaking at 150 rpm in 24 h. The graph legend shows the colony morphology of *Enterobacter* spp. on nutrient agar and the cell morphology under light microscope at 1000× magnification.
(PDF)

S2 Fig Qualitative assay of siderophore, biosafety plates and EPS on *Enterobacter* spp.. (PDF)

S3 Fig Organic acid profiling of NBRIP and Aleksandrov media inoculated with *Enterobacter* spp. using HPLC. (PDF)

S1 Table Closest strains to *Enterobacter* spp. in NCBI GenBank database. (PDF)
