# Supplementary figures and images for: Seed biopriming with P- and K-solubilizing Enterobacter hormaechei sp. improves the early vegetative growth and the P and K uptake of okra (Abelmoschus esculentus) seedling
Source: PLoS One. 2020 Jul 9;15(7):e0232860. doi: 10.1371/journal.pone.0232860 (PMC7347142; doi:10.1371/journal.pone.0232860)

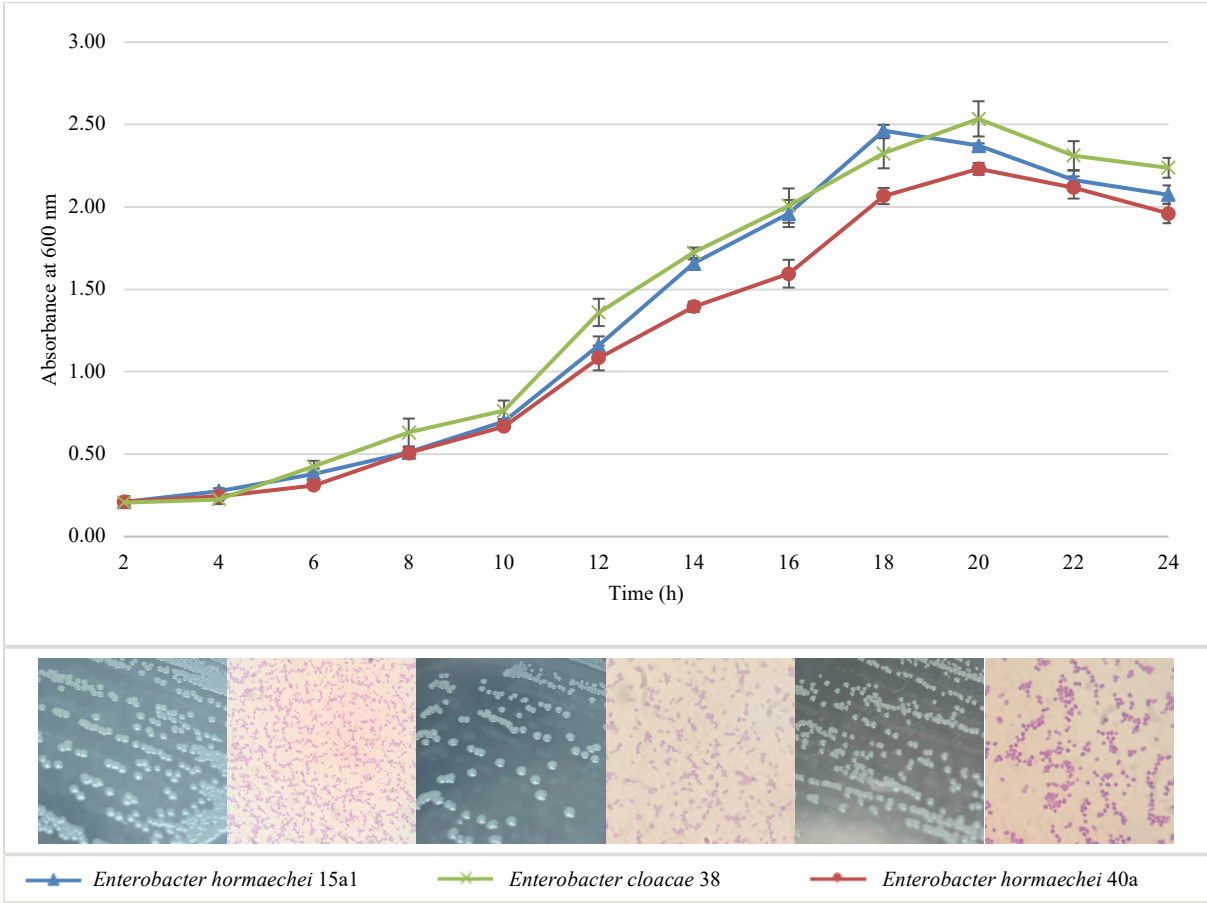

Supplement: S1 Fig — The graph legend shows the colony morphology of Enterobacter spp. on nutrient agar and the cell morphology under light microscope at 1000× magnification. (PDF) [file pone.0232860.s001.pdf]

1    **Supporting information**

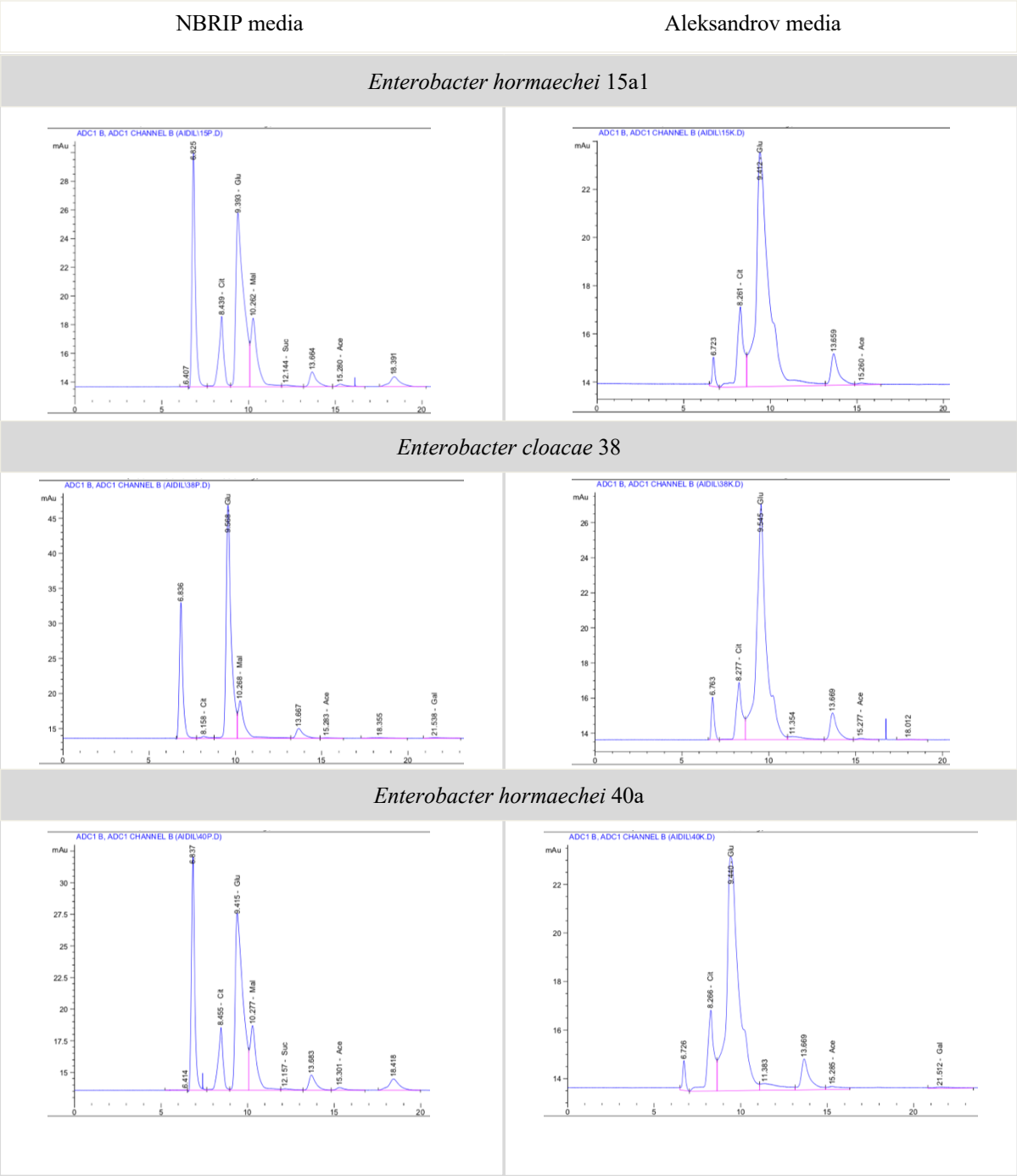

2  
3  
4

Supplement: S3 Fig — (PDF) [file pone.0232860.s003.pdf]
