# Supplementary material for: Seed biopriming with P- and K-solubilizing Enterobacter hormaechei sp. improves the early vegetative growth and the P and K uptake of okra (Abelmoschus esculentus) seedling
Source: PLoS One. 2020 Jul 9;15(7):e0232860. doi: 10.1371/journal.pone.0232860 (PMC7347142; doi:10.1371/journal.pone.0232860)

Supporting information

Siderophore production	γ-haemolysis	EPS production

Enterobacter hormaechei 15a1

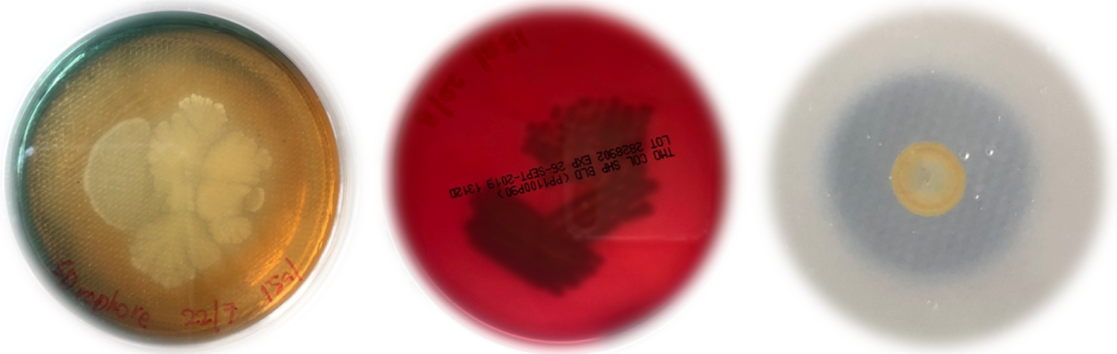

Enterobacter cloacae 38

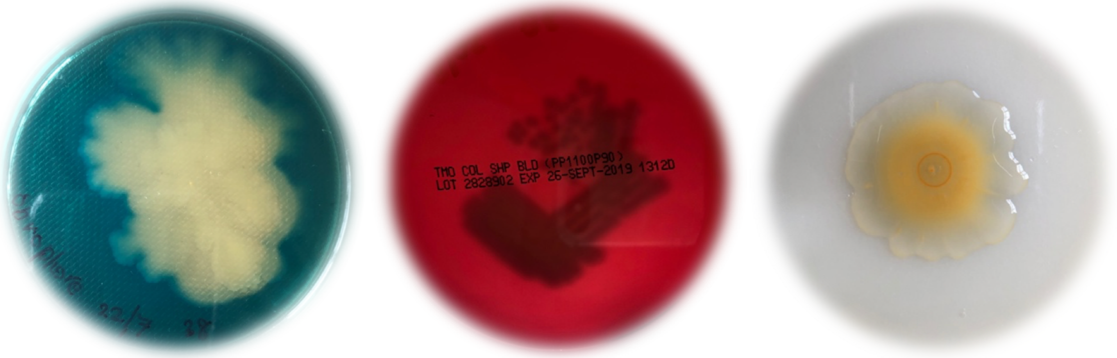

Enterobacter hormaechei 40a

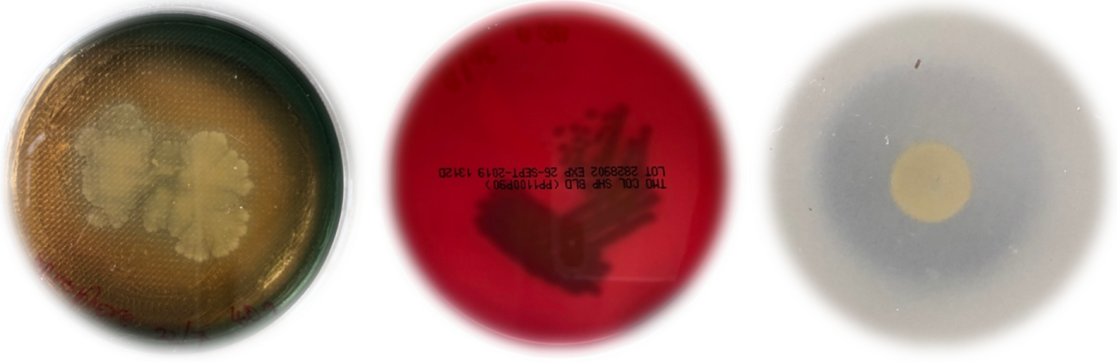

Supplement: S2 Fig — (PDF) [file pone.0232860.s002.pdf]
