# Supplementary material for: Seed biopriming with P- and K-solubilizing Enterobacter hormaechei sp. improves the early vegetative growth and the P and K uptake of okra (Abelmoschus esculentus) seedling
Source: PLoS One. 2020 Jul 9;15(7):e0232860. doi: 10.1371/journal.pone.0232860 (PMC7347142; doi:10.1371/journal.pone.0232860)
Supplement: S1 Table — (PDF) [file pone.0232860.s004.pdf]

Supporting information

Strain	Closest NCBI strain	% Query cover	E-value	% Similarity	Accession
Enterobacter hormaechei 15a1 (MN294583.1)	*Enterobacter hormaechei* strain CPO 4.200	99	0.0	100.00	MN733028.1
	Enterobacter hormaechei strain VITJS3A	99	0.0	100.00	MN258703.1
	Enterobacter spp. BSP6	100	0.0	99.93	KF360068.1
	Enterobacter spp. BSP12	100	0.0	99.93	KF360063.1
	Enterobacter spp. JJDP1	100	0.0	99.93	JQ726698.1
Enterobacter cloacae 38 (MN294584.1)	*Enterobacter cloacae* strain BAB-2824	99	0.0	100.00	KF535159.1
	Enterobacter cloacae strain TLS20	99	0.0	99.93	MH010051.1
	Enterobacter cloacae strain SBP-8	99	0.0	99.93	KJ950709.2
	Enterobacter cloacae strain NIBSM_OsR9	99	0.0	99.93	KY930709.1
	Enterobacter cloacae strain NIBSM_OsL2	99	0.0	99.86	KY927847.1
Enterobacter hormaechei 40a (MN294585.1)	*Enterobacter hormaechei* strain CPO 4.200	99	0.0	100.00	MN733028.1
	Enterobacter hormaechei strain VITJS3A	99	0.0	100.00	MN258703.1
	Enterobacter spp. BSP6	100	0.0	99.93	KF360068.1
	Enterobacter spp. BSP12	100	0.0	99.93	KF360063.1
	Enterobacter spp. JJDP1	100	0.0	99.93	JQ726698.1
